# Mechanistic and Structural Insights into a Divergent PLP-Dependent l-Enduracididine Cyclase from a Toxic Cyanobacterium

**DOI:** 10.1021/acscatal.3c01294

**Published:** 2023-07-11

**Authors:** Jennifer L. Cordoza, Percival Yang-Ting Chen, Linnea R. Blaustein, Stella T. Lima, Marli F. Fiore, Jonathan R. Chekan, Bradley S. Moore, Shaun M. K. McKinnie

**Affiliations:** † Department of Chemistry and Biochemistry, University of California, Santa Cruz, California 95064, United States; ‡ Center for Marine Biotechnology and Biomedicine, Scripps Institution of Oceanography, University of California, La Jolla, California 92093, United States; § Center for Nuclear Energy in Agriculture, University of São Paulo, Piracicaba, São Paulo 13416-000, Brazil; ∥ Department of Chemistry and Biochemistry, University of North Carolina at Greensboro, Greensboro, North Carolina 27402, United States; ⊥ Skaggs School of Pharmacy and Pharmaceutical Sciences, University of California, San Diego, California 92903, United States

**Keywords:** biosynthesis, pyridoxal-5′-phosphate (PLP), enzyme mechanism, noncanonical amino acids, cyanobacteria

## Abstract

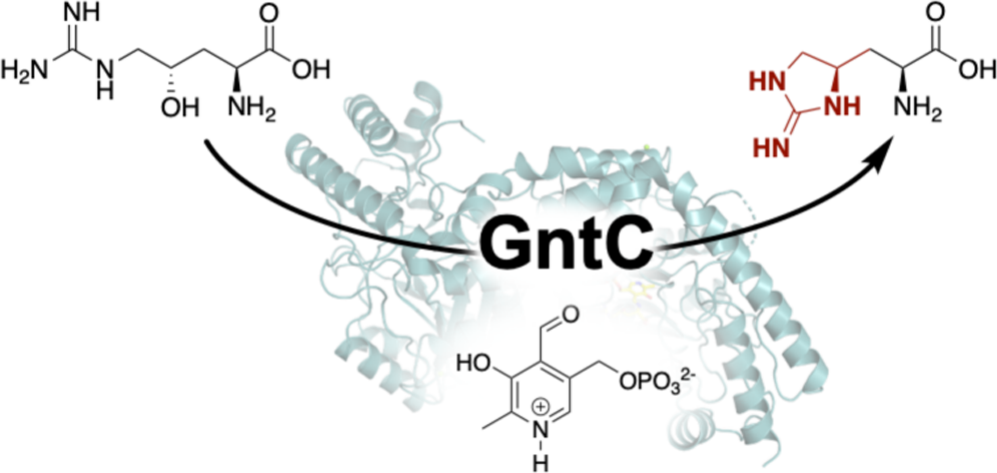

Cyclic arginine noncanonical amino acids (ncAAs) are found in several actinobacterial peptide natural products with therapeutically useful antibacterial properties. The preparation of ncAAs like enduracididine and capreomycidine currently takes multiple biosynthetic or chemosynthetic steps, thus limiting the commercial availability and applicability of these cyclic guanidine-containing amino acids. We recently discovered and characterized the biosynthetic pathway of guanitoxin, a potent freshwater cyanobacterial neurotoxin, that contains an arginine-derived cyclic guanidine phosphate within its highly polar structure. The ncAA l-enduracididine is an early intermediate in guanitoxin biosynthesis and is produced by GntC, a unique pyridoxal-5′-phosphate (PLP)-dependent enzyme. GntC catalyzes a cyclodehydration from a stereoselectively γ-hydroxylated l-arginine precursor via a reaction that functionally and mechanistically diverges from previously established actinobacterial cyclic arginine ncAA pathways. Herein, we interrogate l-enduracididine biosynthesis from the cyanobacterium *Sphaerospermopsis torques-reginae* ITEP-024 using spectroscopy, stable isotope labeling techniques, and X-ray crystallography structure-guided site-directed mutagenesis. GntC initially facilitates the reversible deprotonations of the α- and β-positions of its substrate before catalyzing an irreversible diastereoselective dehydration and subsequent intramolecular cyclization. The comparison of *holo*- and substrate-bound GntC structures and activity assays on site-specific mutants further identified amino acid residues that contribute to the overall catalytic mechanism. These interdisciplinary efforts at structurally and functionally characterizing GntC enable an improved understanding of how nature divergently produces cyclic arginine ncAAs and generate additional tools for their biocatalytic production and downstream biological applications.

## Introduction

**Figure 1:**
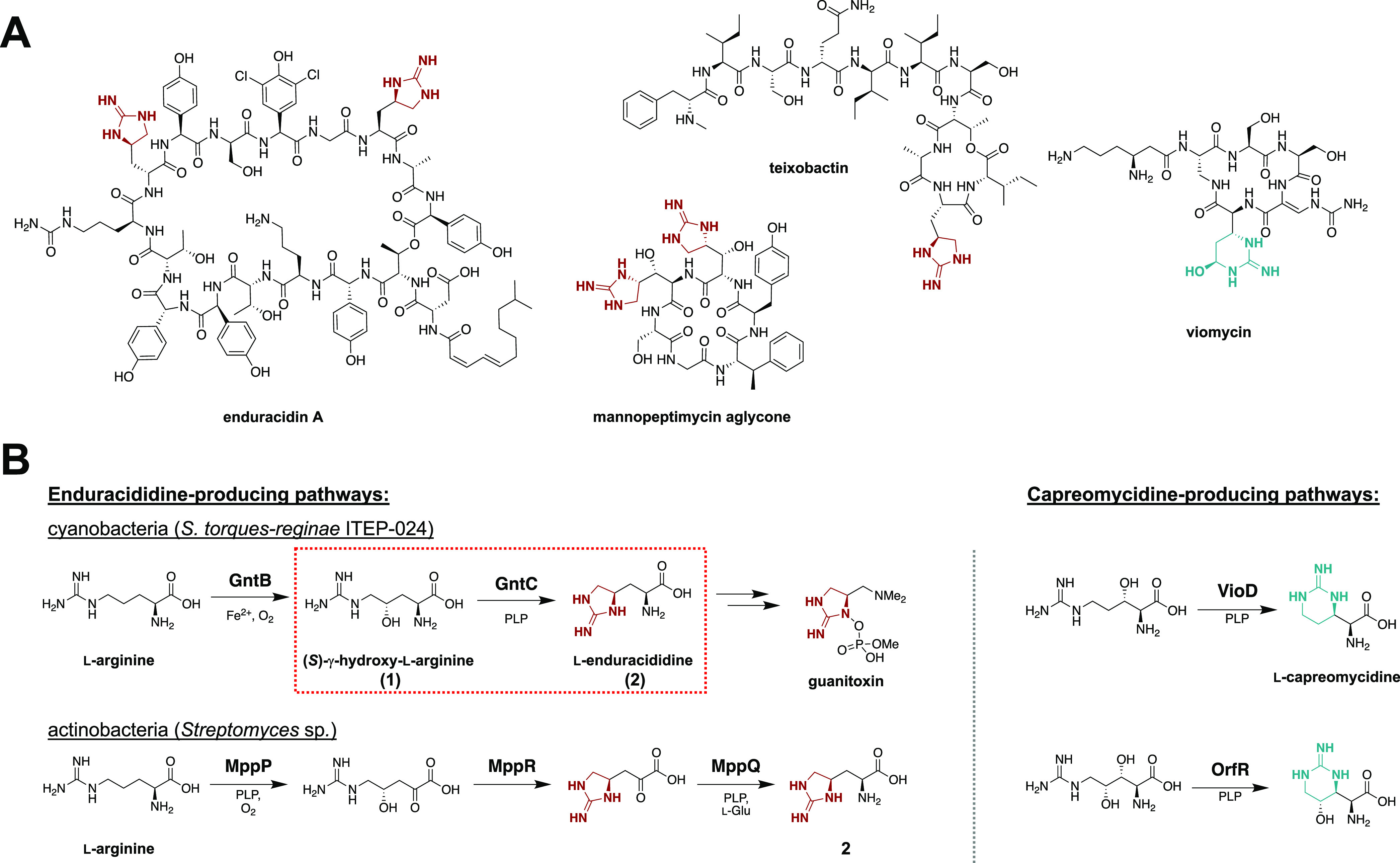
(A) Bioactive antimicrobial nonribosomal peptides containing enduracididine (red) or capreomycidine (blue) cyclic arginine noncanonical amino acids (ncAAs). (B) Characterized biosynthetic strategies toward the construction of cyclic arginine enduracididine (left) and capreomycidine (right) ncAAs. Guanitoxin biosynthesis in cyanobacterium *S. torques-reginae* ITEP-024 proceeds through cyclic arginine intermediate l-enduracididine (**2**) via the cyclodehydration activity of PLP-dependent enzyme GntC. Pyridoxal-5′-phosphate (PLP) enzymology involved in cyclic arginine biosynthesis from actinobacterial mannopeptimycin (MppP, MppQ), viomycin (VioD), and streptolidine (OrfR) pathways.

Cyclic arginine noncanonical amino acids (ncAAs) are relatively rare in nature but are highly represented in bioactive natural products.^1,2^ Traditionally isolated via bioactivity-guided fractionation efforts, antimicrobial nonribosomal peptides like teixobactin,^3^ enduracidin,^4^ mannopeptimycin,^5^ and viomycin^6^ possess these cyclic ncAAs within their chemical structures (Figure 1A). Two isomeric forms currently exist wherein the guanidine moiety is cyclized at either the γ- or β-position of the arginine backbone, creating either the five-membered enduracididine or six-membered capreomycidine scaffolds, respectively. Once constructed, enduracididine and capreomycidine ncAAs can be directly incorporated into nonribosomal peptide synthetase (NRPS) products via specific adenylation domain activation^7^ or further transformed by oxidative enzymes^8,9^ into other cyclic guanidine-containing natural products. Recent advances have shown that capreomycidines can be divergently biosynthesized on NRPSs via dehydrogenation and thioester-mediated Michael addition reactions in the faulknamycin and muraymycin biosynthetic pathways.^10,11^

Currently, cyclic arginine ncAAs have limited commercial availability due to their multiple synthetic steps and heavy reliance on protecting groups.^2^ This has hampered an understanding of their role in establishing the bioactivities of their cognate NRPS products. Advances in small-molecule biocatalysis, particularly in ncAA biosynthesis, have created ample opportunities to repurpose these enzymes for their efficient and scalable construction.^12,13^ Moreover, identifying novel methodologies for the biosynthetic construction of cyclic arginine ncAAs would serve as valuable genome mining hooks to discover new natural products with these moieties.

Recently, we discovered the biosynthetic pathway of guanitoxin, a potent freshwater neurotoxin from the cyanobacterium *Sphaerospermopsis torques-reginae* ITEP-024 which possesses a cyclic guanidine moiety in its highly polar structure (Figure 1B).^14^ Previously named anatoxin-a(s),^15^ guanitoxin exerts its neurotoxicity through the irreversible inhibition of acetylcholinesterase within the peripheral nervous system.^16^ If consumed via contaminated water, guanitoxin can lead to an overexcitation of cholinergic neurons and downstream muscle spasms, respiratory arrest, and potentially death. Within the nine-enzyme biosynthetic pathway that transforms l-arginine into the mature neurotoxin, the pyridoxal-5′-phosphate (PLP)-dependent enzyme GntC constructs the key cyclic guanidine moiety upon which the anticholinesterase *O*-methylphosphate pharmacophore is assembled. Specifically, GntC performs a cyclodehydration of substrate (*S*)-γ-hydroxy-l-arginine (**1**) to l-enduracididine (**2**), cleanly inverting the stereochemistry at the γ-position. This reaction is highly diastereoselective under *in vitro* enzyme assay conditions following 1-fluoro-2,4-dinitrophenyl-5-l-alanine amide (l-FDAA, Marfey’s reagent) derivatization and ultra performance liquid chromatography mass spectrometry (UPLC-MS) analysis. In our initial characterization of GntC, we failed to observe any enzymatic production of *allo*-l-enduracididine diastereomer (**2′**) nor did we observe any conversion of substrate epimer (*R*)-γ-hydroxy-l-arginine (**1′**) within our limits of detection.^14^ While the GntC reaction occurs relatively early in the guanitoxin biogenesis, the biochemical validation was foundational for establishing the biosynthetic pathway. Both **1** and **2** were previously isolated from guanitoxin-producing *Anabaena flos-aquae* cyanobacteria, and the incorporation of backbone-deuterated **1** into the mature toxin was supported via *in vivo* feeding experiments.^17,18^ Although these stable isotope experiments directly implicated **1** in guanitoxin biogenesis, an intriguing reintroduction of hydrogen atoms at the former β-position implied that additional deprotonation/reprotonation chemistry was involved in toxin production (Figure S1).^18^ Following full pathway characterization,^14^ one of these β-position reprotonations could be justified using the retro-aldol (GntG) and transamination (GntE) reactions identified in this study. However, we propose that a mechanistic interrogation into GntC-mediated cyclization of **1** could help further rationalize this *in vivo* result.

PLP is a widely utilized cofactor that facilitates a diverse range of enzyme-catalyzed chemistry, including transaminations, decarboxylations, and aldol reactions.^19^ PLP-dependent enzymes frequently perform stereo- and regioselective reactions under ambient conditions to produce various molecules, including synthetically challenging ncAAs.^20^ The biosynthesis of **2** has previously been characterized in the actinobacterial mannopeptimycin pathway and employs two PLP-dependent enzymes (MppP, MppQ) and an acetoacetate decarboxylase-like superfamily homolog MppR to analogously generate this cyclic ncAA (Figure 1B).^21−24^ PLP-dependent oxidase MppP converts l-arginine to a γ-hydroxylated α-keto acid intermediate,^22,23^ upon which MppR uses an active site lysine to facilitate a stereoselective five-membered ring cyclization while retaining the α-keto acid.^21^ A final PLP-dependent transamination by MppQ completes the biosynthesis of **2**.^24^ In contrast, **2** production in cyanobacterium *S. torques-reginae* ITEP-024 employs transmembrane enzyme GntB to generate linear **1**, which is directly cyclized by PLP-dependent GntC. In addition to the functional differences between the two biosynthetic routes to **2**, GntC has low sequence homology to any of the mannopeptimycin enzymes (30, 34, 5% sequence similarities to MppP, MppQ, MppR, respectively), suggesting a divergent mechanistic and biochemical strategy toward constructing this ncAA.

At a functional level, GntC represents a unique fusion of characterized PLP-dependent enzymes from other biosynthetic pathways that transform amino acid-derived substrates. VioD and OrfR, from the viomycin^25,26^ and streptolidine^9^ pathways, respectively, perform an intramolecular cyclization of the guanidine side chain at the β-position of a hydroxylated arginine precursor, generating the six-membered ring within the capreomycidine ncAA (Figure 1B). Intermolecular nucleophilic addition at the γ-position of a vinylglycine ketimine intermediate is precedented in PLP-dependent enzymes like cystathionine-γ-synthase (CGS) from methionine primary metabolism.^27^ This ketimine intermediate has also been observed in specialized metabolite biosynthetic pathways, including recently characterized fungal enzymes CndF,^28^ Fub7,^29^ and AnkD,^30^ actinobacterial Mur24 involved in antibiotic nucleoside muraymycin biogenesis,^31^ and indirectly through hydration in canavanine catabolism in select Gram-negative proteobacteria.^32^ Although it remains to be biochemically characterized, LolC from the loline biosynthetic pathway is believed to proceed through an analogous ketimine intermediate with an l-proline nucleophile.

Overall, due to its unique sequence homology, divergent chemical reaction, and important role in guanitoxin biosynthesis, we sought to determine the mechanism of GntC-mediated cyclization, rationalize its strict diastereoselectivity, and understand structural features responsible for its catalytic function. Through this investigation into how nature convergently biosynthesizes **2** in phylogenetically distinct microbes, we can better understand the role of this ncAA in divergent natural products and accelerate the biocatalytic application of GntC toward the construction of complex cyclic ncAAs.

## Results and Discussion

### GntC PLP Dependency

To fuel our investigation, substrate (**1**, **1′**) and product (**2**, **2′**) diastereomers were synthesized following previously established synthetic protocols.^14,33^*Sphaerospermopsis torques-reginae* ITEP-024 GntC was heterologously expressed as an *Escherichia coli* codon-optimized N-terminal His_6_-tagged construct and purified to near homogeneity using Ni-NTA affinity chromatography.^14^*In vitro* GntC assays were setup in potassium phosphate (KPi) buffer, amine-containing components were derivatized with l-FDAA, and reaction mixtures were analyzed by UPLC-MS as previously reported.^14^ Because GntC co-purifies with its requisite PLP cofactor, we had yet to firmly confirm its dependency in **2** production. Treatment of purified GntC with PLP inhibitor hydroxylamine followed by buffer exchange abolished any catalytic activity with **1** in the *apo*-GntC form (Figure S2). Reintroduction of two molar equivalents of PLP to the apoenzyme restored **2** production, validating the cofactor dependence of the intramolecular cyclase GntC.

### GntC *In Vitro* Assay Optimization

We initially investigated a range of pHs in KPi buffer to identify ideal conditions for **2** production. Under a 20:1 substrate/enzyme ratio, relative product formation was compared to an l-FDAA-derivatized glycine standard to correct for any derivatization discrepancies, and we determined that GntC behaved optimally at pH 8.0 (Figure S3). Previous reports identified that divalent metal cations can aid PLP-dependent enzyme catalysis through the promotion of electron displacement,^28^ and we wanted to determine if a similar observation occurred with GntC. Counterintuitively, **2** production was not enhanced in the presence of any 1 mM divalent metal salts; instead, incubation with chelator EDTA showed comparable activity to wild-type GntC. Most other ionic additives (Mg^2+^, Ca^2+^, Mn^2+^, Fe^2+^, Zn^2+^, Co^2+^) showed similar or reduced *in vitro***2** formation, with the addition of Cu^2+^ completely abolishing production (Figure S4). We finally investigated the impact of the His_6_ tag on *in vitro***2** production, given the significance of the N-terminus on homodimerization, shaping the active site pocket and establishing catalysis in other PLP-dependent enzymes.^34,35^ We recloned GntC as a C-terminal His_6_ construct and found a slight reduction in catalytic activity compared to the original N-terminal His_6_ construct (Figure S5). In spite of additional optimization efforts, *in vitro* GntC assays were done under identical reaction conditions to those previously described.^14^ Following the development of an l-FDAA-derivatized **2** standard curve (Figure S6), we determined the steady-state kinetic parameters of GntC toward substrate **1** (*k*_cat_ = 0.0061 ± 0.008 min^–1^, *K*_M_ = 150 ± 80 μM (Figure S7)).

### GntC Spectroscopy

**Figure 2:**
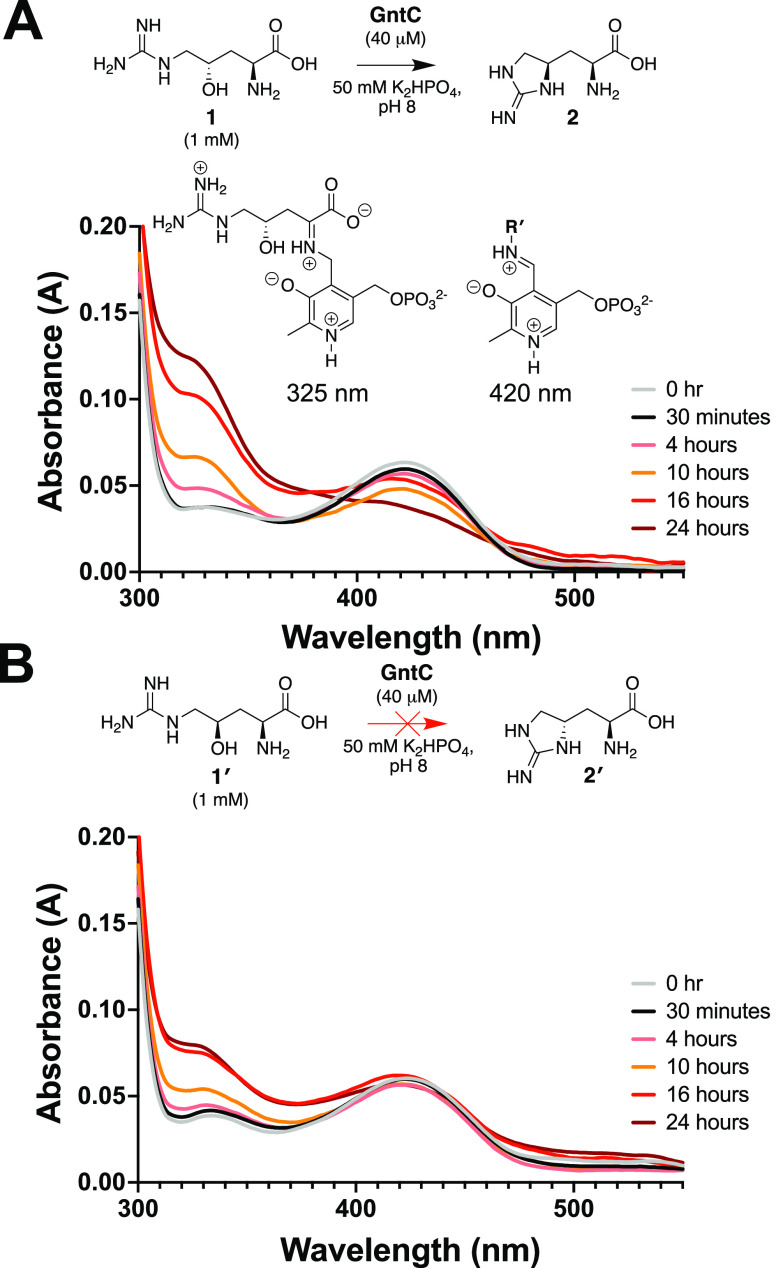
*In vitro* GntC assays with (A) native substrate **1** and (B) diastereomer **1′** show divergent UV–visible absorption spectra at 325 and 420 nm, corresponding to the ketimine and aldimine intermediates, respectively. *R*′ represents either a conserved lysine residue (internal aldimine) or an amine substrate (external aldimine).

Spectroscopic analyses have been performed on various PLP-dependent enzymes and their conjugated cofactors to identify diagnostic quinonoid intermediates in rate-limiting steps of the catalytic cycle,^36−38^ including methionine biosynthetic enzyme CGS^36^ and threonine aldolase enzymes.^39^ Following the incubation of GntC and substrate **1**, we did not observe any extended quinonoid intermediates. Instead, we identified a time-dependent accumulation of an intermediate with an absorbance at 325 nm at the expense of the resting PLP cofactor state at 420 nm (Figures 2A and S8A). The 325 nm chromophore represents the ketimine intermediate^40,41^ and is mechanistically formed from protonation of the quinonoid at the 4′ position. This is generally observed in canonical primary metabolic PLP-dependent aminotransferases and methionine biosynthetic enzyme CGS. Although spectroscopic features consistent with a quinonoid intermediate were not directly observed, we believe that GntC uses a transient quinonoid en route to the ketimine intermediate that accumulates over the *in vitro* time-course experiment. Intriguingly, when substrate diastereomer **1′** was mixed with GntC under identical reaction conditions, a modest increase of this 325 nm intermediate was observed with minimal perturbations to the PLP system itself, even after prolonged incubation (Figures 2B and S8B). These data imply that an extended conjugated state is not involved in the rate-limiting step of the GntC mechanism and that the substrate hydroxyl group stereochemistry plays an important role in not only product output but also spectroscopic features of catalysis. We also incubated product **2** with GntC over 16 h and identified spectroscopic features similar to those following **1** and **1′** reactions (Figure S9). This implies that both ketimine and external aldimine intermediates form at the beginning and end of the GntC mechanistic cycle.

### Mechanistic Studies

**Figure 3:**
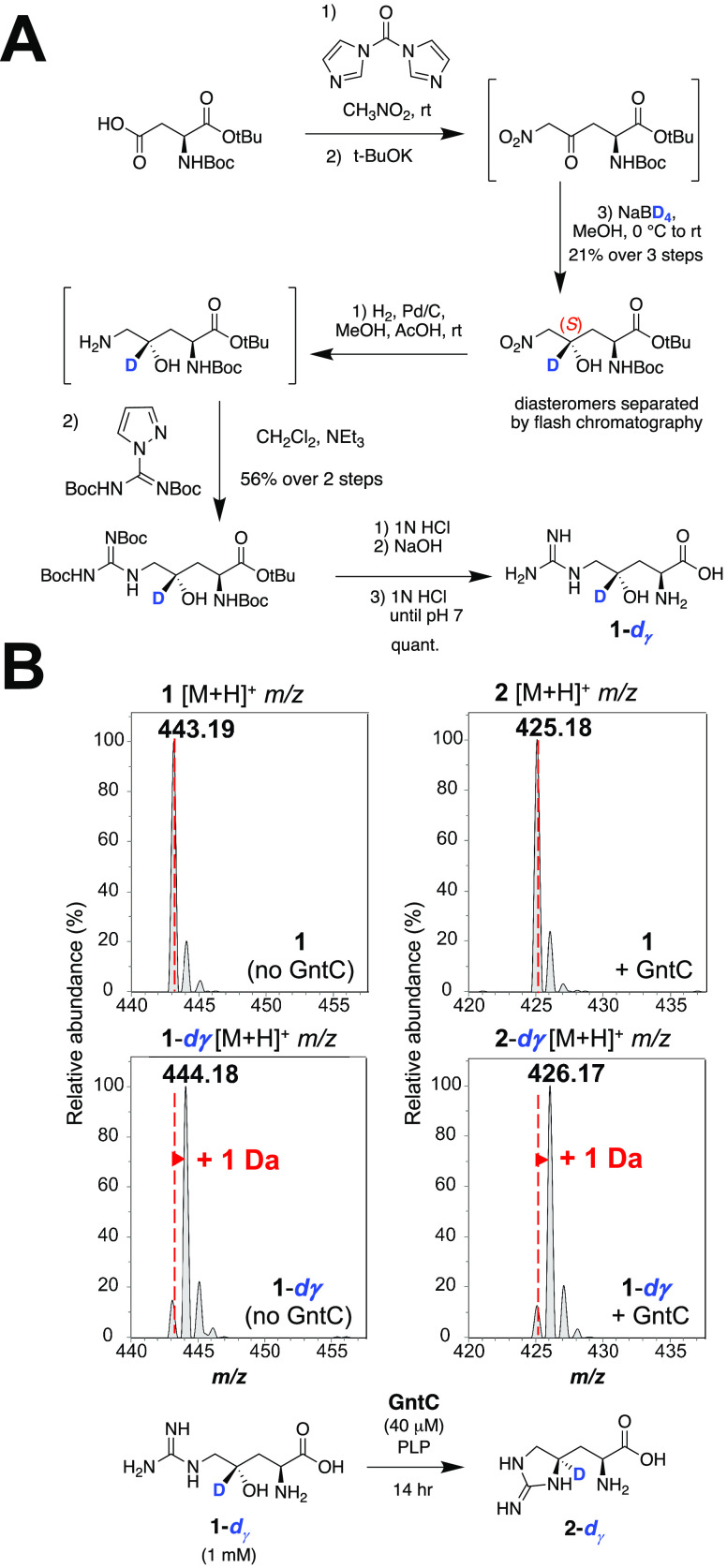
Selective deuteration reveals that GntC does not deprotonate the γ-position of substrate **1**. (A) Synthesis of selectively γ-deuterated substrate **1**-*d*_γ_. (B) UPLC-MS spectra for l-FDAA-derivatized substrates (**1** and **1**-*d*_γ_; left) and products (**2** and **2**-*d*_γ_; right) in the absence and presence of *in vitro* GntC. The increase of +1 Da in product **2-***d*_γ_ supports that the γ-deuterium atom is retained throughout the GntC mechanism.

To assess the mechanism of enduracididine synthesis by GntC, we initially investigated whether any deprotonation/reprotonation or oxidation events occur at the γ-position of substrate **1**. We modified our established synthetic scheme to selectively reduce the *in situ* ketone intermediate with sodium borodeuteride and install one deuterium atom at the γ-position with 95% deuterium enrichment (Figure 3A).^14^ The deuterated (*S*)-alcohol was resolved from its (*R*)-diastereomer via established silica flash chromatography conditions,^14^ then subsequent nitro reduction, guanylation, and deprotection reactions generated the desired γ-deuterated substrate (**1-***d*_γ_). *In vitro* incubation of **1-***d*_γ_ with GntC followed by l-FDAA derivatization and UPLC-MS analysis showed that the resultant cyclic product (**2-***d*_γ_) was one additional Da heavier than derivatized **2** (Figure 3B) and mirrored the isotopic distribution of synthetic substrate **1**-*d*_γ_. The retention of the γ deuterium atom suggests that no deprotonations occur at this position during the GntC mechanism.

**Figure 4:**
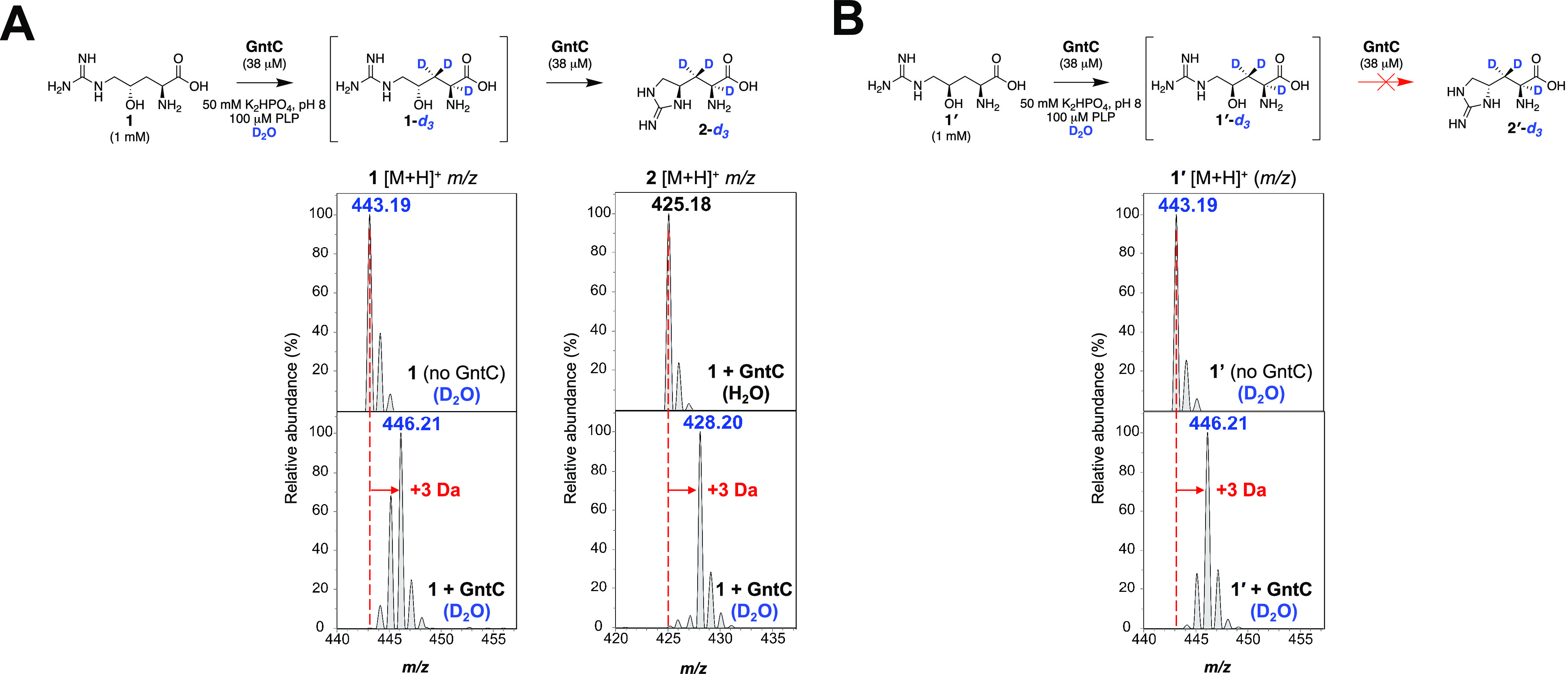
UPLC-MS analyses of l-FDAA-derivatized *in vitro* GntC D_2_O assays identify the incorporation of up to three deuterium atoms in γ-hydroxy-l-arginine substrates. (A) Increase of +3 Da is observed in linear substrate **1** and cyclic product **2** (**1**-*d*_3_ and **2**-*d*_3_, respectively) in the presence of D_2_O and GntC. (B) Substrate diastereomer **1′** also shows an increase of +3 Da (**1′**-*d*_3_) in the presence of D_2_O-buffered GntC despite any cyclic product formation.

To complement the **1**-*d*_γ_ assay, we next aimed to identify which substrate hydrogen atoms were reversibly solvent exchanged during the GntC mechanism via buffered deuterium oxide (D_2_O) assay conditions and mass spectrometry analyses. After exchanging GntC into deuterated KPi buffer following previous literature examples,^42,43^ we employed *in vitro* enzymatic assays under 100% D_2_O conditions. Following the assay, l-FDAA derivatization and UPLC-MS analyses were conducted in regular protic solvents to ensure that only deuterium atoms incorporated to the carbon backbone were retained. Moreover, assays were compared to controls with exogenous PLP, however, omitting GntC to correct for nonenzymatic deuterium incorporation. Analyses of *in vitro* D_2_O reactions identified an increase of 3 Da in product **2** (**2-***d*_3_) in the presence of GntC (Figure 4A), implying that up to 3 deprotonation/reprotonation events occur during the catalytic mechanism. A similar 3 Da increase was observed in substrate **1** (Figure 4A) and inactive diastereomer **1′** (Figure 4B) in the presence of deuterated GntC. This was intriguing as **1′** failed to generate any observable products by UPLC-MS following incubation with GntC.^14^ For both linear **1** and **1**′ substrates, substantial 1 and 2 Da isotopologues were observed following these experiments. These singly and doubly deuterated intermediates could be attributed to partial completion of the GntC reaction and give insight into the overall mechanism. In combination with the **1-***d*_γ_ assay results, these data suggested that D incorporations occurred at the α and β carbons within the GntC active site.

**Figure 5:**
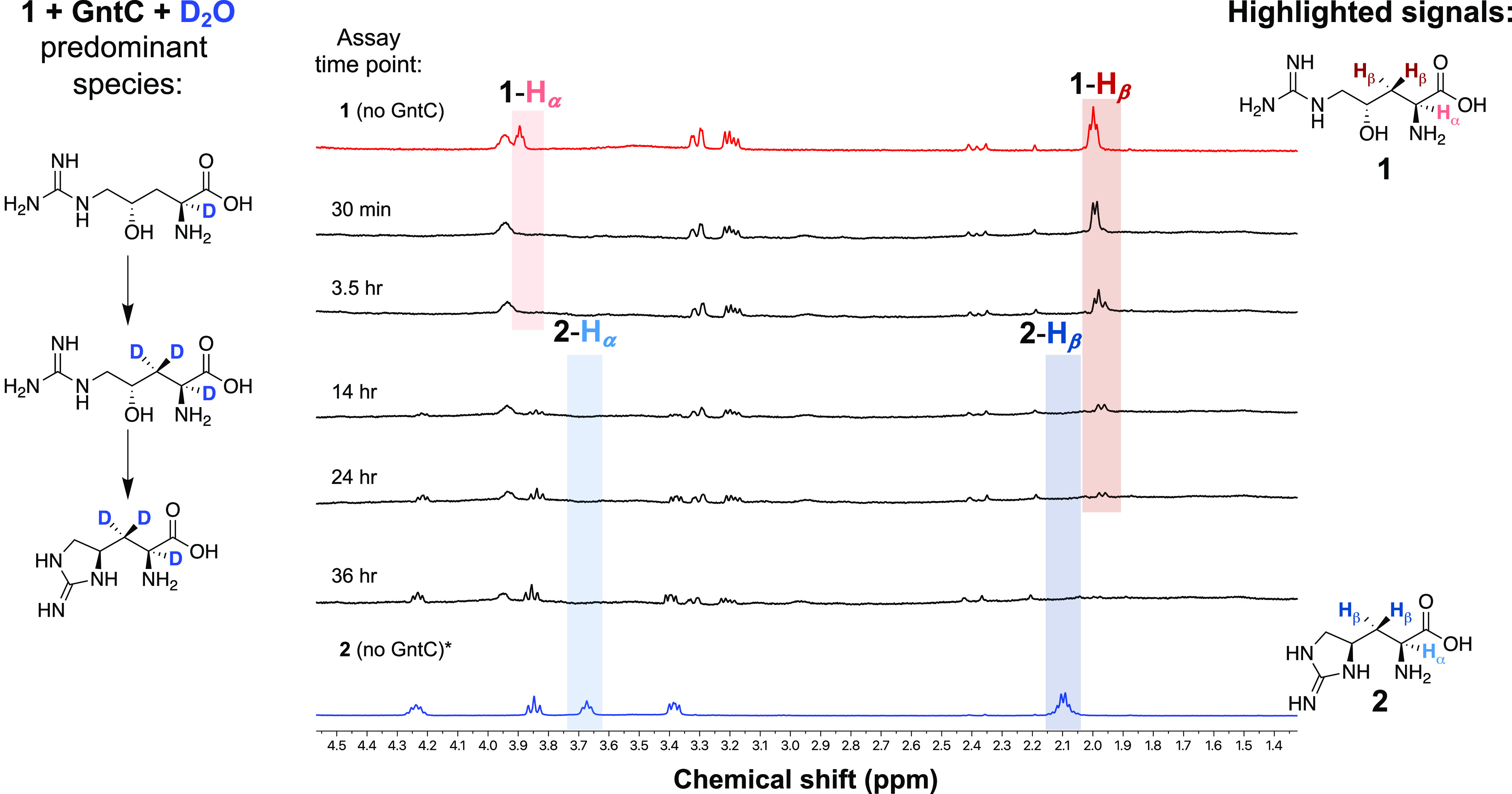
^1^H NMR time-course experiments under D_2_O conditions identify that both the α- and β-hydrogen atoms of substrate **1** and product **2** are solvent exchanged during *in vitro* GntC assays. The predominant chemical species are depicted to the left of GntC reaction time points and compared to **1** (top, red trace) and **2** (bottom, blue trace) standards.

To orthogonally corroborate these results, we employed ^1^H NMR to conclusively identify which hydrogen atoms were solvent exchanged through the course of the GntC reaction. We initially established our assay in protic solvent conditions under a diluted 100:1 substrate/enzyme ratio within a 0.5 mm NMR tube. Following a 14-h reaction end point, we identified the diagnostic α-hydrogen (δ 3.69) and β-hydrogen (δ 2.09) signals that mirrored the synthetic **2** standard (Figure S10). After exchanging GntC into KPi-buffered D_2_O and employing analogous *in vitro* conditions, we tracked reaction progression and D incorporation over time via ^1^H NMR. Under D_2_O conditions, the α-hydrogen of **1** (δ 3.92) was lost within 30 min, while the β-hydrogen signals (δ 1.94) began to change in multiplicity over the course of the reaction, finally diminishing after 24 h (Figure 5). There was an aberrant decrease in one of the multiplexed diastereotopic β-hydrogens, implying a facial preference for this deprotonation. We rationalized this observation by GntC-catalyzed β-hydrogen abstraction, followed by nonstereoselective reprotonation with deuterium oxide. This reversible D incorporation slowly erodes both β-hydrogen signals over time and explains the +2 and +3 Da isotopologues observed following UPLC-MS analyses (Figure 4). Consistent with the loss of **1** hydrogen signals, characteristic **2** signals at δ 4.24, 3.85, and 3.38 appeared after the 14 h mark in the ^1^H NMR time course, with the notable omission of α- and β-hydrogen atoms. Cumulatively, these results support the 3 Da increase under D_2_O conditions and support that deuterium atoms were present at the α- and β-hydrogens within **2**. A similar trend was observed when interrogating substrate diastereomer **1′**, in which α- and β-hydrogen signals were lost in the presence of D_2_O-exchanged GntC, however, with the notable absence of any dehydrated or cyclic products (Figure S11). This provides further insight into the GntC mechanism by suggesting that α- and β-deprotonations occur before the dehydration and cyclization steps necessary for **2** formation. Moreover, this implies that the stereospecific recognition of the γ-hydroxy group by GntC enables the elimination to proceed.

Many PLP transaminases and aldolases exhibit a level of reversibility which can regenerate the starting materials depending on the equilibrium or metabolic flux of the system. To assess the overall reversibility of the GntC reaction, we incubated product **2** with GntC in buffered D_2_O conditions and analyzed the reaction mixture by UPLC-MS after 14 h. While we did observe an increase in 3 Da in product **2** (Figure S12), we did not identify any substrate **1** with any degree of D incorporation nor any other l-arginine-derived products (Figure S13). To further assess the reversibility of the dehydration step, we incubated GntC with substrate **1** under KPi-buffered H_2_^18^O. Following a 14-h assay, UPLC-MS analyses of l-FDAA-derivatized **1** showed no difference in the isotopic distribution in the presence or absence of GntC, implying that heavy water is not being incorporated (Figure S14). This reaction deviates from that of the streptolidine PLP-dependent OrfR capreomycidine cyclase, in which H_2_^18^O was reversibly incorporated into its β-hydroxylated arginine substrate.^9^ These cumulative results show that the GntC-catalyzed cyclodehydration of substrate **1** is unidirectional and that the dehydration is an irreversible mechanistic step under the physiological conditions tested.

In summary, our heavy isotope labeling experiments indicate that up to three deprotonations occur at the α- and β-positions of substrate **1**, these occur before the elimination of the γ-hydroxyl group and cyclization, and the dehydration and overall GntC reaction is not reversible under *in vitro* conditions. Moreover, these results highlight a divergence of this cyanobacterial PLP-dependent arginine cyclase enzyme in contrast to its actinobacterial orthologs. Given the D_2_O results of the substrate diastereomer **1′**, we hypothesized the enzyme active site was responsible for the diastereoselectivity of GntC and next pursued enzyme structural studies to further understand the GntC mechanism and the biochemical source of this selectivity.

### GntC Sequence Homology to PLP-dependent Cyclases

After gaining mechanistic insights into GntC catalytic activity, we compared its amino acid sequence with validated PLP-dependent arginine cyclases to bioinformatically determine any conserved residues that may contribute to the overall mechanism (Table S1). In addition to the active site lysine (K219) that binds the PLP cofactor, sequence alignments of GntC to PLP-dependent capreomycidine cyclases VioD^25,26^ and OrfR^9^ revealed a conserved glutamate residue (E9) and two consecutive serine residues (S25, S26) toward the N-terminus of all three enzymes (Figure S15). OrfR and VioD shared a slightly higher percent sequence similarity to GntC (38 and 44%, respectively) than other PLP-dependent enzymes. Outside of PLP-dependent cyclases, we also compared the amino acid sequence of GntC to functionally analogous PLP-dependent enzymes that perform divergent intermolecular γ-substitutions (CndF, LolC, CGS, Figure S16). Additionally, as GntC bioinformatically annotates as a type I/II PLP-dependent aminotransferase, we independently compared its sequence to *E. coli* AspC (UniProt ID: P00509). The subset of PLP-dependent intermolecular γ-substitution enzymes and aminotransferases displayed comparatively lower (21–31%) amino acid sequence similarities. CGS exhibited the highest percent similarity to GntC and shared residues for PLP anchoring such as the conserved lysine (K219) for internal aldimine formation and aspartate (D186) for pyridine ring nitrogen protonation (Figure S16). These observations suggest there are minimal similar residues at a sequence level between GntC and other γ-substitution enzymes that would bioinformatically contribute to product conversion.

### GntC Crystal Structure and Mutagenesis

**Figure 6:**
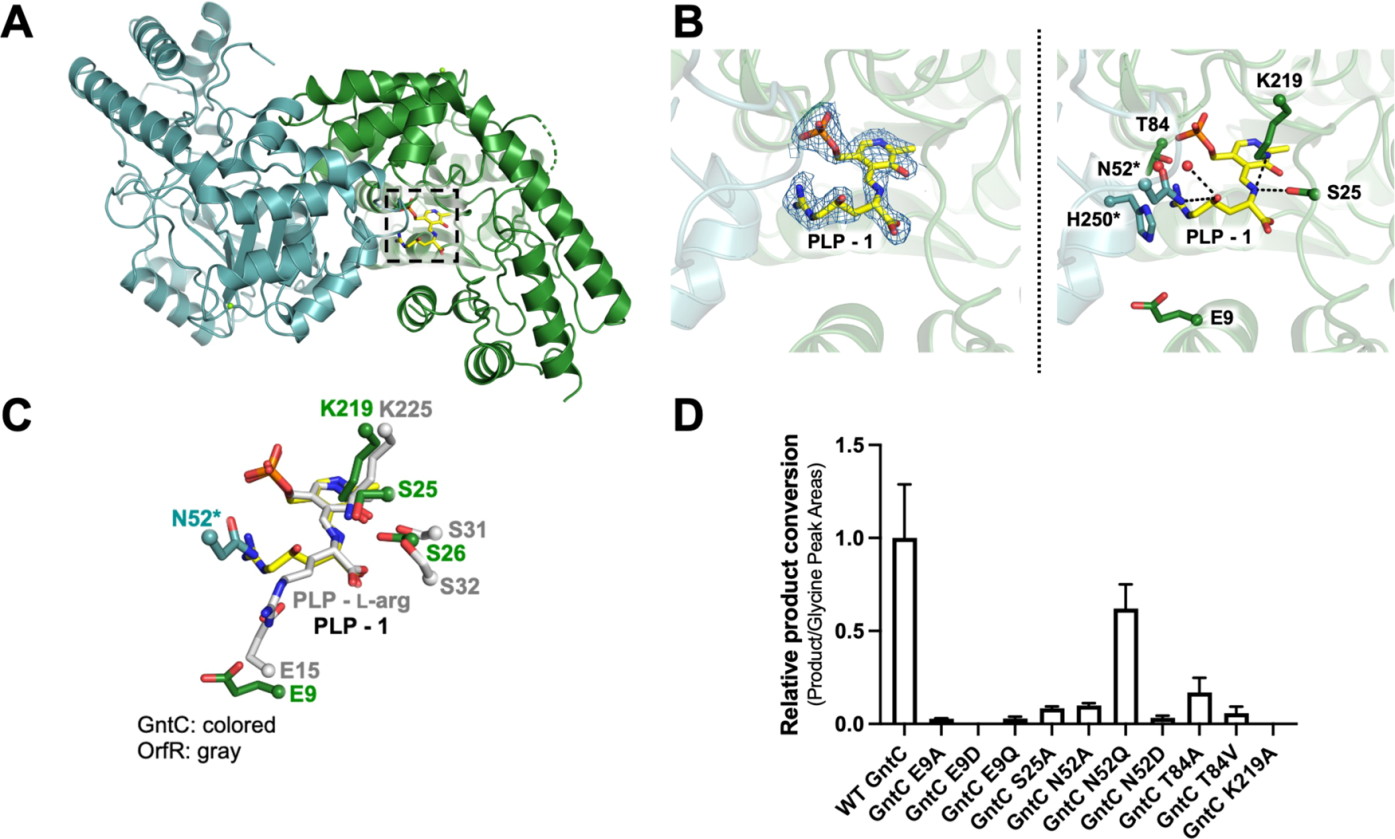
GntC X-ray crystal structure and site-directed mutagenesis identify important amino acid residues for catalysis. (A) GntC crystal structure with bound PLP-**1** substrate (yellow) crystallized as a dimer of homodimers at 2.04 Å resolution (PDB ID: 8FFU, only one homodimer depicted for simplicity). The active site is at the interface of the homodimer between the green and teal monomers, consistent with other PLP-dependent enzyme structures. (B) GntC active site with Fo-Fc electron density map for bound PLP-**1** (blue mesh, σ = 1.0) imposed on the ligand (left) and amino acid residues proposed to participate in substrate recognition and catalysis (right), including E9, S25, T84, and K219 (green monomer), and N52* and H250* (teal monomer). (C) Simplified active site comparison of GntC (green/teal) and capreomycidine cyclase OrfR residues (gray, PDB ID: 4M2M) with bound PLP-**1** (yellow) and PLP-l-arg (gray) substrates, respectively. (D) *In vitro* enzyme assays for **2** production with soluble GntC mutants in comparison to wild-type (WT) GntC.

To gain further insight into the structural features that facilitate catalysis, we obtained crystal structures of GntC with bound PLP (PDB ID: 8FFT). Following heterologous expression and purification, GntC crystals were obtained using a hanging drop method and the structure was solved to a 2.10 Å resolution using molecular replacement with a PLP-dependent aminotransferase from *Streptococcus suis* (PDB ID: 3OP7, 29% sequence identity to GntC) (Figure S17A and Tables S2, S3). GntC crystallized as a dimer of homodimers and adopted a largely α-helical structural fold. PLP was bound to K219 in each monomer as an internal aldimine; this was consistent with sequence alignment results that suggested this lysine was responsible for anchoring the cofactor in the active site.^44^ We subsequently setup individual crystallization trials with PLP-bound GntC and each available substrate (**1**, **1′**) and product (**2**, **2′**) diastereomer. Only **1** successfully cocrystallized with GntC (80% occupancy in one of the monomers) and this external aldimine-containing structure (PLP-**1**) was solved to 2.04 Å resolution (PDB ID: 8FFU) (Figure 6A). PLP-bound and PLP-**1** GntC structures were highly similar (0.31 Å root-mean-square deviation (RMSD) for backbone atom overlap) with the major structural difference being an additional N-terminal ordered helix that helps form the active site in the PLP-**1-**bound complex (Figure S17B and Tables S2, S3). When comparing PLP-bound and PLP-**1** internal and external aldimine substrates, there was a 30° downward rotation of the pyridine ring, orienting the amino acid substrate toward a new ordered N-terminal helix that was not present in the first structure (Figure S17C). Consistent with many type I/II PLP-dependent enzymes,^19,44^ the GntC active site is found at the homodimer interface. Although it is predominantly composed of residues from one of the monomers, important contributions from the corresponding homodimer occur on at least one face of the active site pocket.

We next sought to identify GntC active site residues within hydrogen-bonding distances of the PLP-**1** adduct that could contribute to catalysis and diastereoselectivity (Figure 6B). Conserved active site K219 lies 2.8 Å from the α-amine forming the external aldimine, T84 is located 2.4 Å away from the PLP phosphate and 3.3 Å away from the guanidinium moiety of **1**, H250 putatively shapes the guanidinium binding pocket, and S25 is within close proximity to the α-carboxylate. Given the mechanistic importance of the γ-hydroxyl group of **1**, we searched for residues of interest near this moiety to help dictate the diastereospecificity of GntC cyclization. The only amino acid side chain in proximity was N52, which was 4.1 Å away and contributed from the other subunit in the dimer (defined as N52* herein). Other residues involved in PLP binding (Figure S17D,E) include D186 (2.8 Å from the pyridoxal ring nitrogen), H188 (2.8 Å from the PLP phosphate group), N158 and Y189 (both within 3 Å of the PLP hydroxyl group), and F108 (π stacking with PLP cofactor). Additionally, R346 was positioned 2.9 Å from the α-carboxylate of substrate PLP-**1** and presumably contributes to amino acid substrate recognition. GntC requires an active site base to support the α- and β-deprotonation results obtained; based on the PLP-**1** structure, the best candidate is K219.

When the crystal structures of GntC and OrfR (PDB ID: 4M2M) were aligned (1.7 Å RMSD for overlap), S25 and S26, K219, and E9 in the GntC crystal structure overlaid with OrfR residues identified by sequence alignment (Figure 6C). These analogous amino acid residues were previously biochemically validated to have important mechanistic roles in the OrfR-catalyzed PLP-dependent formation of γ-hydroxylated capreomycidine ncAA.^9^ Briefly, the two continuous serine residues (OrfR S31 and S32) coordinate with active site lysine (OrfR K225) to facilitate the initial α-deprotonation of dihydroxylated l-arginine via an activated water molecule, and the conserved glutamate residue (OrfR E15) putatively helped tune the nucleophilicity of the guanidine before cyclization. Given the similar functions of the two enzymes, we hypothesized that these residues could serve similar purposes in GntC. However, the divergent cyclic ncAA connectivity and heavy isotope incorporation mechanistic differences fail to provide conclusive identification to the source of γ-hydroxy recognition and diastereoselectivity through this OrfR structural comparison.

The most chemically analogous enzymatic reaction to GntC is arguably MppR, which catalyzes the intramolecular stereoselective cyclization en route to **2** biosynthesis.^21^ However, MppR significantly diverges as it does not use a PLP cofactor and works upon keto acid-containing starting materials and products. Unsurprisingly, very minimal amino acid sequence or structure alignment similarities (PDB ID: 4JME) were observed between these topologically distinct enzymes with different quaternary structures. Negligible active site homology exists between these two enzymes, and additional comparisons were not pursued despite the similar cyclization functions. Although it does not participate in arginine cyclization biochemistry, we performed a structure alignment of GntC to known crystal structure of type I PLP-dependent enzyme aspartate aminotransferase *E. coli* AspC^45^ (PDB ID: 1ARS); this analysis revealed few conserved residues outside of those needed for PLP binding activity and α-carboxylate recognition (Figure S18).

Outside of known protein crystal structures, we also aligned GntC to AlphaFold 2.0 models^46^ of VioD^25,26^ and Fgm3^47^ from the viomycin and fusaoctaxin biosynthetic pathways, respectively. VioD catalyzes the PLP-dependent formation of a six-membered capreomycidine ring analogous to OrfR but with a singly β-hydroxylated l-arginine precursor. Structural alignment with modeled VioD supported the role of previously identified GntC amino acid residues (K219, R346, S25, S26, E9, F108) implicated in PLP-**1** interaction and catalysis (Figure S19). Unique similarities to the GntC and VioD comparative model were R227 and Y189; however, both interact with the PLP cofactor instead of the bound substrate. Lastly, Fgm3 is a PLP-dependent enzyme that uses **1** as a substrate to catalyze a retro-aldol reaction.^47^ Aligning modeled Fgm3 and GntC identified the conservation of active site K219 and T84 that coordinates with the guanidine group of **1** (Figure S20). Fgm3 contains a histidine residue instead of F108 in GntC for π stacking above the pyridine ring of PLP. Intriguingly, Fgm3 has a tyrosine residue (Y225) in proximity to the γ-hydroxyl group of PLP-**1** that is absent in GntC. Y225 in Fgm3 putatively could serve as the general base needed for the retro-aldol activity of this enzyme and biochemically rationalize the divergent chemistries these two enzymes exhibit upon the same substrate.

Using these cumulative structural comparisons, we selected six residues of interest hypothesized to contribute to GntC catalysis (E9, S25, N52, T84, K219, and H250). We generated site-specific alanine mutants at these six positions and additionally prepared GntC variants with more conservative mutations to address specific features about that amino acid side chain. E9D and E9Q were engineered to investigate the impact of chain length and hydrogen-bond donor ability of this previously established catalytic residue on OrfR, T84V and T84S were targeted to assess if pocket shaping or hydrogen bonding are important for guanidinium recognition, and N52D and N52Q mutants were designed to probe the impact of hydrogen-bond donation ability and chain length respectively on putative γ-hydroxyl recognition and elimination (Table S4). GntC mutants were heterologously expressed and purified using previously described procedures except H250A and T84S which were insoluble and not pursued further. Given the proposed influence of homodimerization on shaping the GntC active site and role of N52* in γ-hydroxyl recognition, all purified mutants were assessed via analytical size exclusion chromatography and displayed comparable levels of dimerization to wild-type GntC (Figure S21).

The five soluble GntC alanine mutants (E9A, S25A, N52A, T84A, K219A) were assayed for **2** production in triplicate and normalized to wild-type GntC using a 500 μM glycine internal standard following l-FDAA derivatization and UPLC-MS analysis (Figure 6D). In all cases, a reduction of **2** formation was observed, indicating the importance of these residues in GntC catalysis. We hypothesized that K219A would disrupt the external aldimine resting state of GntC and remove the covalent tether retaining PLP in the active site. The complete loss of **2** production *in vitro* validated that K219 is an essential residue. Mutating nearby S25 to an alanine reduced **2** production by an order of magnitude, suggesting that organization within this area is beneficial for GntC catalysis. E9 mutation also showed a substantial loss of **2** production regardless of the amino acid residue mutation (E9A, E9D, E9Q). This glutamate residue was identified from the sequence alignment of OrfR and VioD and was hypothesized to help the cyclization mechanism. Within GntC, E9 is 7.7 Å away from the hydroxyl group in the PLP-**1-**bound structure, so we propose that it plays a stabilizing role in the second-shell coordination of the substrate or construction of the active site. The T84A and T84V mutants were designed to disrupt any hydrogen-bonding interactions that might coordinate the incoming guanidinium nucleophile; decreased **2** production with both mutants provides support for this claim. Finally, the N52 mutant series provided the largest variations in **2** formation. The N52A mutant reduced product by approximately 10-fold, with even a further decrease by the N52D mutation. However, we observed a significant retention of activity with the N52Q mutant, suggesting that the amide side chain of N52 plays an important role in GntC catalysis. We hypothesize that the amide hydrogen bonds with the γ-hydroxyl of **1** to make it a better leaving group during the GntC-catalyzed dehydration step of the mechanism either directly or via a hydrogen-bonded network of water. Although the N52 side chain is 4.1 Å away from the γ-hydroxyl within the PLP-**1-**bound GntC structure, there could be additional conformational flexibility during catalysis or substrate reorientation following α- and β-deprotonations. The reduction of **2** production during the N52A and N52D mutants further supports this hypothesis (Figure S22A,B). Amide-containing amino acid residues have been found to play important catalytic roles in PLP-dependent enzymes, such as Q52 in dialkylglycine decarboxylase, which facilitates favorable stereoelectronic interactions during the reaction.^48^ These data give insight into the inner biochemical workings of GntC catalysis and enable its biocatalytic application toward ncAA syntheses.

### GntC Mechanistic Proposal

**Scheme 1:**
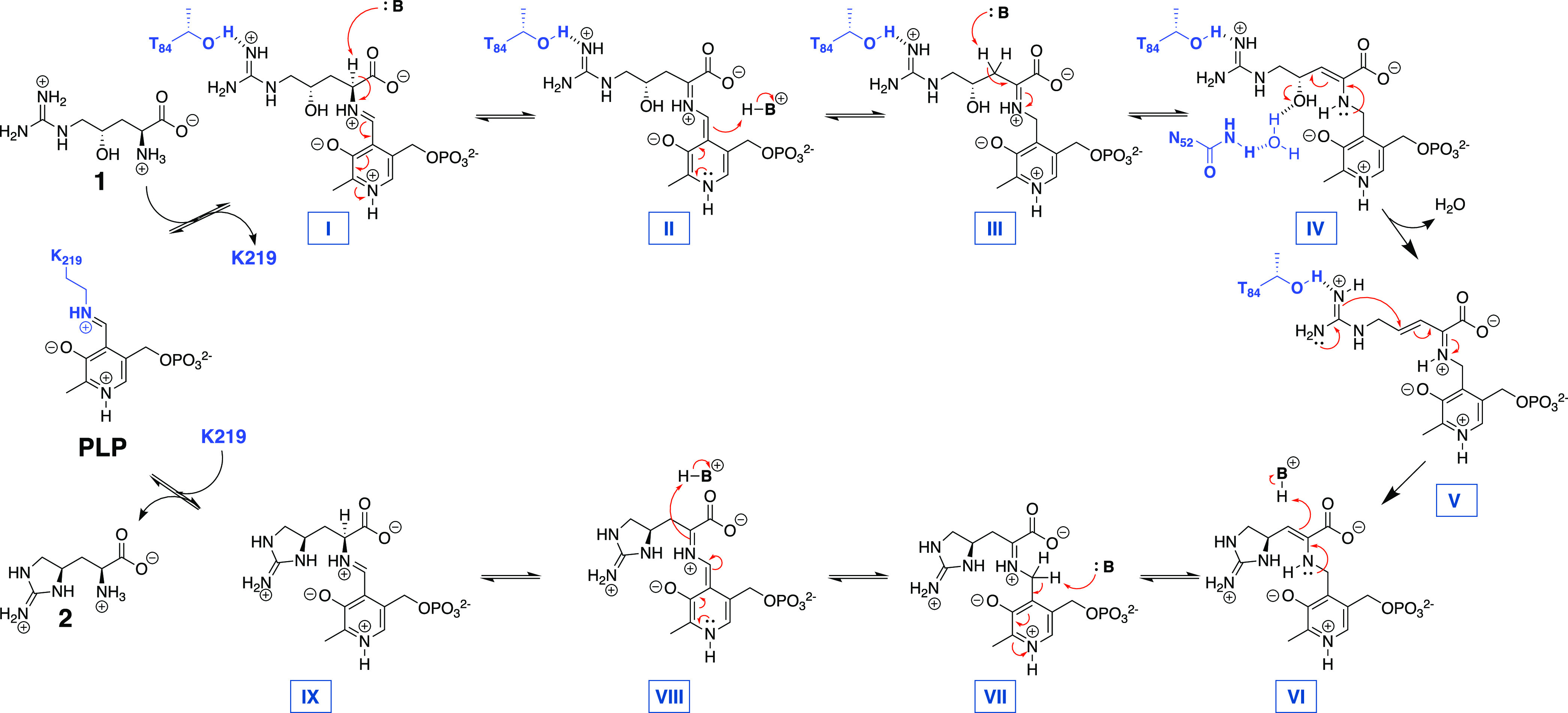
Proposed GntC Mechanism Based on Cumulative Spectroscopic, Heavy Isotope Labeling, NMR, and X-ray Crystallography and Site-Directed Mutagenesis Results

Based on cumulative spectroscopic, UPLC-MS, NMR, and X-ray crystallography-derived site-directed mutagenesis results, we have proposed the following GntC mechanism (Scheme 1). Initially, substrate **1** forms an external aldimine (I) with the PLP cofactor, displacing the active site K219 tether. Subsequent α-hydrogen deprotonation, either by K219 or by a coordinated water molecule, generates the canonical quinonoid species (II), which can be reprotonated to rearomatize the PLP cofactor and form ketimine III. Reversible deprotonation of the β-hydrogens facilitates the formation of enamine IV. The side-chain amide of N52* next forms a diastereoselective hydrogen bond either directly or through a coordinated water molecule with the γ-hydroxy group to power the irreversible dehydration and subsequent formation of α,β-unsaturated imine intermediate V. Afterward, the T84-coordinated guanidine moiety performs an irreversible intramolecular Michael addition to form the cyclic ring and resultant enamine (VI). Reprotonation at the β-position regenerates ketimine VII, which can be subsequently deprotonated to quinonoid (VIII) and reprotonated at the α-position to form PLP- **2** (intermediate IX). Final replacement with K219 liberates product **2** and regenerates the internal aldimine to complete the catalytic cycle.

While the early and late mechanistic steps are consistent with many canonical PLP-dependent enzymes, the novelty around the hydroxyl group elimination and subsequent intramolecular cyclization are what distinguish this cyanobacterial homolog from its more distant orthologs. Capreomycidine cyclases VioD and OrfR use the nucleophilicity of the quinonoid intermediate to catalyze the β-hydroxyl group elimination, and then adapt the extended conjugation and electrophilicity of the resulting pyridinium cofactor to facilitate the intramolecular guanidine cyclization (Figure S23). The lack of extended chromophores following GntC spectroscopic analyses suggests an absence of quinonoid-containing intermediates proposed to be mechanistically involved in actinobacterial β-hydroxyarginine cyclases. It is possible that a direct substitution could occur with the guanidine nucleophile on intermediate IV to directly form VI, bypassing intermediate V. Characterized intermolecular PLP-dependent γ-substitution enzymes, including CGS, CndF, Fub7, AnkD, Mur24, and putative LolC, follow a Michael-type addition of diverse nucleophiles onto a vinylglycine ketimine derived from a γ-functionalized amino acid substrate (Figure S24A,B). In contrast, GntC sets up ketimine intermediate V with only a γ-alcohol as a leaving group and uses an intramolecular guanidine moiety as a nucleophile instead (Figure S24C). Through the mechanism, GntC uses a γ-substitution strategy (like CndF and CGS) to perform the intramolecular cyclization in the formation of a five-membered ring ncAA (like non-PLP-dependent enzyme MppR). This cyanobacterial biosynthetic strategy represents a hybrid of two different approaches to generate cyclic arginine ncAA **2.**

GntC adds to the repertoire of PLP-dependent enzymes that have been recently discovered that catalyze unconventional reactions. Select examples include BesB from the β-ethynylserine pathway that creates a terminal alkyne;^49^ SbzP which catalyzes a γ-substitution of β-nicotinamide adenine dinucleotide onto *S*-adenosylmethionine in the altemicidin biosynthetic pathway;^50^ Claisen chemistry in saxitoxin^51^ and ketomemicin^52^ biosyntheses; the decarboxylative aldol reaction facilitated by UstD that has seen exceptional biocatalytic application;^53^ and the diverse range of PLP-dependent oxidases that have been uncovered in recent years.^22,54^ Despite the ubiquitous presence of PLP in primary and secondary metabolic enzymes, nature continues to adapt this cofactor in unique ways to generate diverse and biocatalytically useful chemical transformations.

## Conclusions

In conclusion, GntC is the first characterized example of an l-enduracididine cyclase from a cyanobacterium. Through its interdisciplinary interrogation using spectroscopic, stable isotope labeling, NMR, MS, and X-ray crystal structure–function studies, we conclude that GntC follows a distinct mechanism from characterized actinobacterial PLP-dependent cyclases and behaves more like an intermolecular γ-substitution PLP-dependent enzyme. This study seeks to better understand the biosynthesis of cyclic arginine ncAAs from diverse microbial species. Moreover, this improved understanding creates opportunities to apply GntC as a biocatalyst for the scalable production of known and novel ncAAs.

## Supporting Information Available

Crystal structure files for 8FFT (CIF)
Crystal structure files for 8FFU (CIF)
General materials and methods; molecular biology and biochemical methods; enzyme assay methods; protein crystallograpy methods; chemical synthesis; NMR spectra for SI-1, SI-2, and 1-*d*; and Protein Data Bank crystal structure reports for 8FFT and 8FFU (PDF)
All unprocessed NMR files for the synthesis of **1-*****d***_**γ**_ and intermediates (ZIP)

## Supplementary Material

cs3c01294_si_001.cif

cs3c01294_si_002.cif

## Abbreviations

l-FDAA: 1-fluoro-2,4-dinitrophenyl-5-l-alanine amide, Marfey’s reagent
ncAA: noncanonical amino acid
NRPS: nonribosomal peptide synthetase
PLP: pyridoxal-5′-phosphate
RMSD: root-mean-square deviation
